# Zolpidem Maintains Memories for Negative Emotions Across a Night of Sleep

**DOI:** 10.1007/s42761-021-00079-1

**Published:** 2021-11-12

**Authors:** Katharine C. Simon, Lauren N. Whitehurst, Jing Zhang, Sara C. Mednick

**Affiliations:** 1 Department of Cognitive Science, University of California, Irvine, 2201 Social & Behavioral Sciences Gateway, Irvine, CA 92697 USA; 2 Department of Psychology, University of Kentucky, Lexington, KY 40506 USA

**Keywords:** Emotion, Sleep, Memory, Pharmacology

## Abstract

Zolpidem, a common medication for sleep complaints, also shows secondary, unexpected memory benefits. We previously found that zolpidem prior to a nap enhanced negative, highly arousing picture memory. As zolpidem is typically administered at night, how it affects overnight emotional memory processing is relevant. We used a double-blind, placebo-controlled, within-subject, cross-over design to investigate if zolpidem boosted negative compared to neutral picture memory. Subjects learned both pictures sets in the morning. That evening, subjects were administered zolpidem or placebo and slept in the lab. Recognition was tested that evening and the following morning. We found that zolpidem maintained negative picture memory compared to forgetting in the placebo condition. Furthermore, zolpidem increased slow-wave sleep time, decreased rapid eye movement sleep time, and increased the fast spindle range in NREM. Our results suggest that zolpidem may enhance negative memory longevity and salience. These findings raise concerns for zolpidem administration to certain clinical populations.

**Supplementary Information:**

The online version contains supplementary material available at 10.1007/s42761-021-00079-1.

## Introduction

The inability to sleep is a pervasive, cross-cultural public health problem for which treatment can include therapeutic or pharmacological intervention (Morin & Benca, 2012; Peterson & Benca, 2006). One commonly prescribed medication for sleep complaints is the short-acting hypnotic zolpidem, a GABA A agonist, that enhances inhibition throughout the central nervous system resulting in sleep initiation and maintenance (Drover, 2004; Morlock et al., 2006). Administering zolpidem selectively improved cognition, with enhanced performance after being observed for declarative verbal memory but not perceptual learning, and this change correlated with increased spindle density (Mednick et al., 2013). In addition, another study investigated the impact of zolpidem on emotional memory using three levels of valence (positive, neutral, and negative) and two levels of arousal (low and high; Kaestner et al., 2013). They found zolpidem increased memory for emotionally arousing negatively valanced pictures, compared with a placebo and a comparison hypnotic, sodium oxybate (see Melendez et al. 2005 and Hall-Porter et al., 2014 for studies that did not find memory enhancement with zolpidem).

The presence of a sleep-dependent memory enhancement is posited to be due to zolpidem’s inducement of additional sleep spindles (9–15 Hz) during non-rapid eye movement (NREM) sleep (Brunner et al., 1991; Feinberg et al., 2000). Spindles are one candidate mechanism—in conjunction with slow oscillations (< 1 Hz)—supporting the preservation and consolidation of hippocampal-dependent memories during sleep (Mednick et al., 2013). It is not known, however, if zolpidem’s effects on emotional episodic memory extend to a full night of sleep. Given that the recommended usage and typical mode of zolpidem administration are for nocturnal sleep, our current study extends prior research to investigate the impact of zolpidem on emotional memory consolidation over a night of sleep.

We employed a double-blind, placebo-controlled, within-subject, cross-over design to investigate if zolpidem, compared to placebo, preferentially boosts the retention of emotions, compared to neutral, pictures over a night of sleep. All subjects learned neutral and negative pictures in the morning and memory retention was tested 12 h (wake consolidation) and 24 h (wake plus sleep consolidation) after encoding. We specifically focused on negatively valanced, high arousal versus neutral given the prior results (Kaestner et al., 2013). Zolpidem or placebo was administered prior to overnight sleep in the lab. Thus, we could observe changes in memory across a night of sleep with or without drug administration. We hypothesized that ingesting zolpidem prior to a nocturnal sleep episode, compared to placebo, would enhance memory retention for negative compared to neutral memories at the 24-h recognition test the subsequent morning. Furthermore, we predicted that increased sleep spindles in the zolpidem condition would correlate with memory retention for negative pictures, with greater sigma power correlating with better long-term recognition.

## Methods

### Subjects

Thirty-three healthy, non-smoking participants between the ages of 18 and 30 (*M*_age_ = 20.8 years, 19 females) provided informed consent. Our study was approved by the Western Institutional Review Board and the University of California, Riverside Human Research Review Board. We compensated participants monetarily. All subjects were healthy with no personal history of neurological, psychological, or other chronic illness. Our exclusion criterion included the following: irregular sleep/wake cycles, past history or present diagnosis of a sleep disorder, personal or familial history of diagnosed psychopathology, substance abuse/dependence, loss of consciousness greater than 2 min, a history of epilepsy, current use of psychotropic medications, non-correctable visual impairments, or any cardiac or respiratory illness that might affect cerebral metabolism. Participants were assessed for inclusion in-person using (1) a modified Structured Clinical Interview (SCID) for the Diagnostic and Statistical Manual of Mental Disorders-IV (DSM-IV) and (2) study-physician approved self-report questionnaires examining current and past health and wellness (First et al., 1997). After initial inclusion, all participants underwent a medical evaluation with the study physician and were given a toxicology screening for schedule I and II drug substances. Additionally, subjects included were naïve to or had limited contact with (< 2 lifetime use and no use in last year) zolpidem. No adverse events due to pharmacological administration were reported by participants during the study. Lastly, the descriptive sleep outcomes reported herein share a subset of data with sleep outcomes that have been reported in additional papers (Tselha et al., 2019; Zhang et al., 2020; Whitehurst et al., 2019).

### Drug Protocol

We administered a single 10-mg dose of zolpidem (zolpidem), a positive allosteric modulator of GABAA receptors with a short half-life (1.5–4.5 h) and rapid onset which was prepared by the MDMX Corona Research Pharmacy. Zolpidem is a GABAA agonist known to depress the central nervous system resulting in reduced sleep latency (Drover, 2004; Farrant & Nusser, 2005). Placebo (placebo) pills were composed of microcrystalline cellulose and contained no active medications. Both zolpidem and the placebo capsules were administered in single encapsulated pills that were visually indistinguishable. Visits were counterbalanced and conducted 1 week apart to allow for drug washout.

### Materials

Stimuli for this task consisted of negative and neutral pictures selected from the International Affective Picture System (IAPS; Lang et al., 1997). We controlled for arousal and valence using the standardized IAPS ratings. We chose pictures that would maximize the differences between neutral, low arousing (valence: *M* = 5.13; *SD* = 1.44; arousal: *M* = 3.87; *SD* = 2.19) and negative, highly arousing (valence: *M* = 2.88; *SD* = 1.57; arousal: *M* = 5.52; *SD* = 2.09) pictures. Of the chosen images, negative and neutral pictures were counterbalanced across condition without repetition. We presented our stimuli on a Windows computer using Matlab^14^ with Psychtoolbox (Kleiner et al., 2007). Our task code (encoding and recognition test) is freely available online at https://github.com/MednickLab/GenericMemoryTask.

### Emotion Picture Task

#### ***Encoding Procedure (See Fig. ******1****** for Schematic)***

**Fig. 1:**
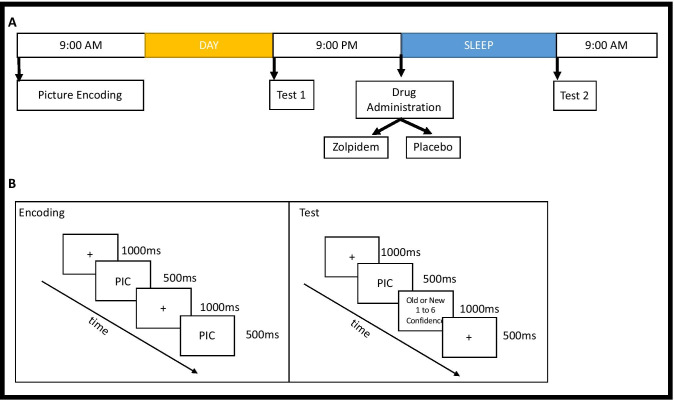
Experimental schematic. **A** The timeline for the paradigm is presented. Subjects were taught pictures in the morning. 12 h later, they were tested (test 1). Subjects were administered either a placebo or zolpidem prior to sleep. Subjects slept in the lab overnight with polysomnographic monitoring. Subjects were tested again the next morning. **B** Learning paradigm. At encoding, subjects were presented with randomized negative or neutral images. At the test, subjects were asked to identify if the images were new or old

At encoding, all subjects viewed pictures for 500 ms with a fixation marker present for 1000 ms prior to picture onset. For each visit, subjects viewed 20 negative and 28 neutral pictures. The extra 8 neutral pictures were split between the first 4 and last 4 pictures viewed to control for primacy and recency effects. After picture offset, subjects responded within a 2000-ms window as to whether the picture was taken indoors or outdoors. Feedback was not provided for the indoor/outdoor rating. Subjects were provided explicit timing for the study using broad language about the memory task but were not given details about the memory task until each of the respective sessions (encoding vs testing). As such, subjects knew they would be trained on material and tested.

#### Recognition Test

Subjects were tested twice, once after 12 h of wake (test 1) and a second time, 24 h later after a period of nighttime sleep (test 2). For both conditions, test 1 and test 2 each consisted of half the pictures encoded (10 negative and 10 neutral) and 20 novel pictures (10 negative and 10 neutral). There was no repetition of the novel distractor pictures from test 1 to test 2. The 8 pictures controlling primacy/recency were not included in analyses. The novel pictures were unique across conditions and tests. For both recognition tests, subjects were presented each picture and prompted to respond with their confidence in recognition on a 1 to 6 scale. The scale ranged from 1 = “very confident the picture was present,” 3 = “not sure, but think the picture was present,” 4 = “not sure, but think the picture was not present,” and 6 = “very confident the picture was not present”. Subjects were required to respond to each picture with no time limit. In between pictures, subjects were shown a 1000-ms fixation cross at the center of the screen.

#### Emotion Picture Analysis

For each subject, we collapsed across confidence ratings and calculated the subject’s responses as “present” for subject responses 1, 2, or 3, or “not present” for subject responses 4, 5, or 6. With this, we then calculated each subject’s accuracy (% of pictures correct) at both test 1 (12-h delay) and test 2 (24-h delay). For both test 1 and test 2, we calculated the discriminability index, *d*’, using the z transform of hit rate (% of pictures present at encoding correctly identified at test; zHitRate) – z transform false alarm rate (% of pictures *incorrectly* identified as present at encoding at test; zFalseAlarmRate). We further calculated the difference scores between test 1 and test 2 (test 2–test 1) to examine performance change across sleep and wake.

### Experimental Design and Protocol

#### ***Experimental Design (See Fig. ******1****** for Schematic)***

We designed our sleep study to understand the impact of zolpidem on overnight emotional memory consolidation compared with a placebo-control. We employed a double-blind, placebo-controlled, within-subject, cross-over design. Subjects slept in the lab overnight with either zolpidem or placebo. Subjects experienced both drug conditions, which were randomized and counterbalanced across subjects. This allowed us to determine the impact of zolpidem vs. placebo on sleep-dependent memory consolidation across a night. Other results comparing the placebo condition and a second, separate drug condition, dextroamphetamine have been presented elsewhere (Whitehurst & Mednick, under review). Data in the current study only used the placebo and zolpidem conditions. For all subjects, we randomized and counterbalanced emotional picture sets.

#### Procedure

Please see Fig. 1 for the experimental design schematic. For the week prior to participation, all subjects completed daily sleep diaries and wore actigraphs to ensure consistent sleep–wake cycles and at least 7 h of sleep each night.

#### Sleep Conditions

All subjects reported to the lab between 8:00 AM and 9:00 AM and were administered the encoding phase of the emotional picture task. After encoding, subjects remained in the lab while undergoing hourly vitals monitoring (blood pressure, heart rate, and subjective measurements) until 2:00 PM. All subjects returned to the lab at 9:00 PM for test 1. Between morning and evening sessions, subjects refrained from exercise, sleep, caffeine, or other drug substances, and were continually monitored by actigraphy. After testing, subjects were administered either a 10-mg dose of placebo or zolpidem. Subjects’ vitals were monitored (blood pressure, heart rate, and subjective measurements) during the polysomnography administration (as discussed below) until lights out at approximately 11:00 PM. Subjects were woken by 9:00 AM and provided breakfast. All subjects were then administered test 2 at approximately 10:30 AM. Subjects experienced this exact timeline twice, once for zolpidem and once for placebo administration.

#### Polysomnography (PSG)

Electroencephalography (EEG) data during sleep was acquired using a 32-channel cap (EASEYCAP GmbH) with Ag/AgCI electrodes placed according to the international 10–20 System (Jasper, 1958). Of the 32 channels, 10 electrodes were used to collect electrocardiogram (ECG), electromyogram (EMG), electrooculogram (EOG), ground, a reference channel (FCz location, retained after re-referencing), and mastoid (A1 & A2) recordings. We recorded EEG with a 1000-Hz sampling rate and, after recording, re-referenced all electrodes to the contralateral mastoid (A1 & A2). We applied low pass (0.3 Hz) and high-pass filters (35 Hz) to all EEG and EOG channels. We preprocessed the data in BrainVision Analyzer 2.0 (BrainProducts, Munich Germany) and removed artifacts and arousals via visual inspection. We scored the raw sleep EEG data according to Rechtschaffen and Kales in 30-s epochs using eight scalp electrodes (F3, F4, C3, C4, P3, P4, O1, O2), EMG, and EOG (Rechtschaffen & Kales, 1968). We calculated minutes in each sleep stage (wake, stage 1, stage 2, stage 3, and REM), sleep latency (SL; minutes between lights out and initial epoch of the sleep stages), total sleep time (TST), wake after sleep onset (WASO), and sleep efficiency (SE).

#### Power Spectrum

To examine the impact of zolpidem on sleep frequency bands, we analyzed the following: slow-wave activity (SWA; 0.3–1 Hz), delta (1–4 Hz), theta (4–7 Hz), slow sigma (9–11 Hz), and fast sigma (12–15 Hz). We rejected EEG epochs contaminated by muscle movement and/or other artifacts using a simple out-of-bounds test (± 200-μV threshold) on a high-pass filtered (0.5 Hz) version of the EEG signals. We computed the EEG power spectra using the Welch method (4-s Hanning windows with 50% overlap) on the artifact-free 30-s epochs. We then estimated the averaged power spectra for both stages 2 and 3 for each subject. We correlated memory scores with averaged SWA from the frontal electrodes (F3 and F4), averaged theta, and slow and fast sigma from the central channels (C3 and C4). These channels were chosen to investigate power given that delta is known to be the most prominent over the frontal lobes, sigma activity most prominent over the central channels, and theta activity over the fronto-central region (Kurth et al., 2010; Mari-Acevedo et al., 2019).

#### Spindle Detection

Our spindle detection methodology is based on Wamsley and colleague’s (Wamsley et al., 2012) methodology (see Malerba et al., 2018; Naji et al., 2019; Satarri et al., 2017, or Zhang et al., 2020 for more details on published results using the same spindle detection). We detected spindle events within stage 2 and stage 3 at central channels C3 and C4. We applied a continuous wavelet transform to the signal with the amplitude (a moving window if 100 ms) being compared to the threshold four times the window’s mean. Identified peaks were considered spindle event peaks. An individual spindle envelope (start to end) was considered from the point in which the amplitude peak crossed then returned below the average threshold.

#### Slow Oscillation-Spindle Coupling (SO-Spindle Coupling)

We first detected slow oscillations within stage 2 and stage 3 using an algorithm with the same criteria as Massimini et al. (2004) and Dang-Vu (2008). Each EEG channel’s signal was first filtered between 0.1 and 0.4 Hz using a zero-phase bandpass. From there, each slow oscillation was then detected using the following criteria: (1) the signal had an initial down zero-crossing followed by an up zero-crossing and then one more down zero-crossing, (2) the down state duration was between 300 ms and 1 s, (3) each event had a maximum length < 10 s, (4) down state amplitudes were ≥ 80 μV, and (5) up state amplitudes were less than 140 μV. We then detected slow oscillation-spindle couplings or temporal coincidence of the events. Spindles that coincided with the SO envelope were considered coupled if they fell within − 1.25 before or 1.25 after the SO down state time (see Satarri et al., 2019 or Zhang et al., 2020 for more details on published results using the same methodology). We separately calculated stage 2 and stage 3 SO-spindle coupling density by dividing the total number of coupled events by the total time spent in the sleep stage.

#### Data Reduction

Six subjects were unable to complete the study design across the sleep conditions due to scheduling conflicts. Two subjects’ EEG were lost due to experimental error and not included in sleep correlations. We also removed 4 subjects’ behavioral data that was 2.5 standard deviations above or below the mean. We report degrees of freedom for each analysis to clarify total subjects within each behavioral and sleep analyses.

### Statistical Analyses

#### Memory Performance

We evaluated subjects’ overall performance using *d*’, which is a measure of memory discrimination that assesses each subject’s sensitivity to discriminate between the correct target picture (hit) and foil (false alarm; Z(hit rate)–Z(false alarm rate)). We separately computed *d*’ for each emotion at both test 1 and test 2. To assess memory performance change across time, we calculated the *d*’ difference (test 2–test 1) for each emotional category. For our within-subject conditions (sleep: zolpidem and placebo), we assessed their emotional memory performance using a 2 × 2 × 2 repeated measures ANOVA with within-subject factors of drug (zolpidem vs placebo) by test (test 1 and test 2) by emotion (neutral vs negative). To account for the different rates of drug absorption due to weight across our subjects, we entered weight (mean centered) as a covariate in each of these analyses. We corrected for multiple comparisons using the Holm-Bonferroni method.

#### Sleep

To examine the impact of zolpidem on nighttime sleep, we ran paired *t*-tests on total sleep time (TST), minutes in each stage (stage 1, stage 2, stage 3, and REM), wake after sleep onset (WASO), and sleep efficiency (SE). We ran paired *t*-tests across the power spectrum (SWA, delta, theta, fast sigma, and slow sigma activity) within sleep stages.

#### Sleep and Memory

We used Pearson’s *r* to assess the relationship between memory performance and power spectra bands within conditions (zolpidem and placebo). We also explored if a relationship existed between memory performance and EEG sleep features, specifically sleep spindle density and SO-coupling density.

## Results

### Memory Performance: Zolpidem vs Placebo

#### Memory Discrimination (d’)

**Fig. 2:**
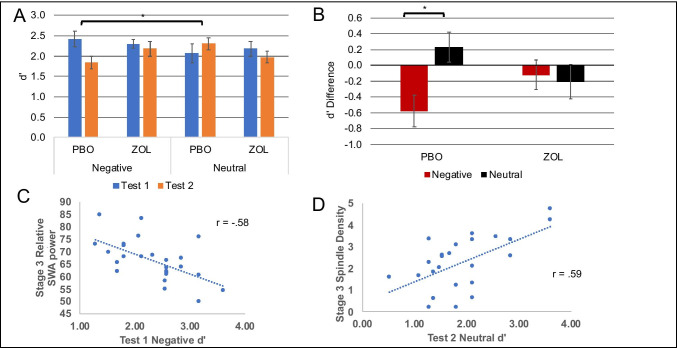
Emotional memory. **A** Memory performance (*d*’) at test 1 and test 2 for placebo and zolpidem conditions. Subjects had greater retention at test 1 than 2 and a significant three-way interaction between drug × test × emotion. **B** Visual demonstration of 3-way interaction here shows the *d*’ differences between test 1 and test 2 for negative and neutral images. Only a significant difference in retention is present in the placebo condition. **C** Stage 3 relative SWA activity negatively correlated with test 1 negative d’ memory in the zolpidem condition. **D** Stage 3 spindle density positively correlated with test 2 neutral *d*’ memory in the zolpidem condition. Both correlations withstood correction, *p* < .0025

We first performed an omnibus 2 × 2 × 2 repeated measures ANOVA using within-subject factors of drug (zolpidem vs placebo) by test (test 1 and test 2) by emotion (neutral vs negative (see Table 1). The ANOVA revealed a significant main effect of test, *F*(1,21) = 7.015, *p* = 0.015, with greater retention at test 1 than test 2 and a significant three-way interaction of drug by time by emotion, *F*(1,21) = 8.954, *p* = 0.007; see Fig. 2.

**Table 1:** Memory performance. Subjects’ memory performance at test 1 and test 2

			Placebo			Zolpidem		
			Mean	*SD*	*d*’ (*SD*)	Mean	*SD*	*d*’ (*SD*)
Test 1	Negative	Hit rate	0.83	0.14	2.43 (1.0)	0.82	0.18	2.3 (.6)
		False alarm	0.17	0.17		0.11	0.14	
	Neutral	Hit rate	0.75	0.18	2.07 (1.23)	0.75	0.16	2.36 (.96)
		False alarm	0.16	0.15		0.11	0.11	
Test 2	Negative	Hit rate	0.71	0.17	1.84 (.82)	0.78	0.18	2.18 (.93)
		False alarm	0.17	0.2		0.15	0.11	
	Neutral	Hit rate	0.7	0.2	2.3 (.73)	0.70	0.16	1.98 (.73)
		False alarm	0.08	0.09		0.11	0.09	

Table represents mean and standard deviation for all performance parameters for both neutral and negative pictures by drug and placebo conditions

To break down this three-way interaction, we first separately analyzed test 1 and test 2 *d*’ performance. For each test, we ran a 2 × 2 repeated measure ANOVA with within factors of drug (zolpidem vs. placebo) and emotion (negative and neutral). At test 1, we found no significant main effects or interactions on memory discrimination (*p*’s > 0.193). In contrast at test 2, we found a significant interaction of drug × emotion, *F*(1, 24) = 5.967, *p* = 0.022 but no main effect of emotion or drug (*p*’s > 0.360). Post hoc analyses with Holm-Bonferroni corrections revealed that the placebo condition showed significantly greater neutral than negative memory discrimination, *t*(25) = 2.721, *p* = 0.012, meaning under placebo, subject’s maintained their memory for neutral pictures while forgetting negative pictures. For the zolpidem condition, there were no significant differences in discrimination performance between the emotional picture categories, *t*(26) = 1.012, *p* = 0.321.

We then evaluated the three-way interaction for both conditions by assessing the change in discrimination performance over time. For each emotion, we subtracted test 2 *d*’ − test 1 *d*’ for a *d*’ difference measure (our 4 dependent variables were ZolNegDiff, ZolNeuDiff, PboNegDiff, PboNeuDiff; see Fig. 2a and 2b). We found significantly reduced memory discrimination for negative but not neutral pictures in the placebo condition, *t*(23) =  − 2.358, *p* = 0.027, whereas the zolpidem condition showed no change in memory discrimination in either memory category, suggesting minimal forgetting over time, *t*(24) = 1.202, *p* = 0.241. Placebo showed greater retention of neutral pictures than the zolpidem condition, *t*(25) = 2.297, *p* = 0.03. However, this difference did not remain significant after correction. Drug conditions did not differ in *d*’ change for negative pictures.

#### Accuracy

Additionally, we performed the same 2 × 2 × 2 repeated measures ANOVA on memory accuracy. We found significant main effects for emotion, *F*(1,24) = 12.458, *p* = 0.002 and test, *F*(1, 24) = 12.013, *p* = 0.002. We did not find any significant interactions (*p*’s < 0.176). Planned comparisons revealed that negative pictures were correctly remembered more so than neutral (*p* = 0.001) across both test 1 and test 2 and that subjects had greater accuracy at test 1 compared to test 2 (*p* = 0.002).

#### False Alarms

We then performed a 2 × 2 × 2 repeated measures ANOVA using within-subject factors of drug (zolpidem vs placebo) by test (test 1 and test 2) by emotion (neutral vs negative) on false alarms. Our analysis revealed a significant main effect of emotion, *F*(1,23) = 7.701, *p* = 0.011, significantly greater false alarms for the negative compared to neutral objects, and a significant three-way interaction of drug by test by emotion, *F*(23) = 4.894, *p* = 0.037. We found no other main effects or interactions (*p*’s < 0.322). Post hoc analyses revealed no significant differences after Holm corrections at test 1 (*p*’s > 0.015). At test 2, the placebo condition had significantly greater false alarms for negative objects than neutral objects, *t*(27) = 3.450, *p* = 0.002.

#### Sleep

General sleep data for the sleep condition is presented in Table 2. There were no significant differences between conditions for total sleep time, stage 1, or stage 2 (*p*’s > 0.441). Compared to placebo condition, administration of zolpidem caused an increased amount of total time spent in stage 3, *t*(27) = 2.162, *p* = 0.04 and, concomitantly, a reduction in total time spent in REM, *t*(27) =  − 2.791, *p* = 0.01. To evaluate changes in relative spectral power between sleep conditions, we first compared SWA and delta in the averaged *F* channels separately for sleep stages 2 and 3 (see Table 3; for absolute spectral power differences, please see supplemental results). We found significantly more relative delta activity in the placebo than zolpidem condition, *t*(27) = 2.403, *p* = 0.017, but no difference in relative SWA. We then evaluated zolpidem’s effect on slow sigma, fast sigma, and theta in the averaged *C* channels separately for sleep stages 2 and 3. Consistent with prior findings (Colvonen, et al., 2019; Kobayashi, et al., 2007; Kurth, et al., 2010), we hypothesized a priori that sigma power would be increased in the zolpidem compared to the placebo condition. Both stage 2 and stage 3, compared to placebo, zolpidem showed significantly greater overall relative fast sigma in the averaged central channels (stage 2: *t*(27) =  − 4.045, *p* < 0.001; stage 3: *t*(27) =  − 2.921, *p* < 0.001).

**Table 2:** Descriptive sleep data is presented. Subjects in the zolpidem compared to placebo condition spent more time in stage 3 and less time in REM

	Placebo		Zolpidem	
	Mean (min)	*SD*	Mean (min)	*SD*
TST	536.48	48.12	538.37	39.67
Stage 1	14.16	8.52	13.625	11.69
Stage 2	282.804	54.36	287.78	47.33
Stage 3	110.82	39.83	121.21*	40.78
REM	128.67*	32.29	115.28	28.48
WASO	31.3	27.6	25.73	26.00
SE	92.39	5.64	93.26	4.88

Table represents mean and standard deviation of each sleep parameter for placebo and zolpidem conditions. Each stage is calculated in minutes. Asterisks represent significance at *p* < 0.05

**Table 3:** Relative power spectra. In stage 2, subjects had greater relative delta and relative theta in the placebo condition, while in both stage 2 and stage 3, subjects had greater relative fast sigma in the zolpidem condition

		Placebo		Zolpidem	
		Mean	*SD*	Mean	*SD*
Stage 2	SWA	59.11	8.68	60.54	10.7
	Delta	13.98*	1.96	13.55	2.32
	Theta	2.62*	.70	2.34	.676
	Slow sigma	.779	.371	.847	.454
	Fast sigma	1.42	.509	1.74*	.735
Stage 3	SWA	62.79	6.30	65.9	7.85
	Delta	16.14	1.96	14.96	2.14
	Theta	1.37	.417	1.24	.445
	Slow sigma	.295	.191	.348	.294
	Fast sigma	.496	.229	.640*	.409

Table represents mean and standard deviation of measurement of relative power across the spectral frequency bands. Asterisks represent significance at *p* < 0.05

#### Sleep and Memory

To explore if the drug-induced changes to sleep impact post-sleep memory performance, we examined the relationships between relative sleep spectral power (SWA, delta, slow sigma, and fast sigma) and memory performance for stage 2 and stage 3 sleep separately (see Fig. 2). Results reported are those that withstood Bonferroni correction (alpha = 0.0025). Upon examination of the zolpidem condition, we found a significant negative relationship between relative SWA power and negative picture *d*’ performance at test 1 in stage 3 at F4 (*r* =  − 0.58, *p* = 0.002), such that subjects with worse memory performance had greater subsequent relative SWA at night. We also found a significant positive relationship in stage 2 with relative fast sigma power at C4 (*r* = 0.60, *p* = 0.0019), such that those with better negative picture test 1 memory performance subsequently had more relative sigma power. We found no relationship between relative spectral power and memory in the placebo condition that withstood correction.

#### EEG Events and Memory

We also ran post hoc exploratory analyses to evaluate the relationship between memory performance and EEG features separately for the zolpidem and placebo conditions. For each sleep stage, we evaluated subjects’ spindle density and SO-spindle coupling density. Results reported are those that withstood Bonferroni correction (alpha = 0.004). In the zolpidem condition, we found a significant positive correlation in stage 3 sleep of C4 spindle density across the night with the morning test 2 neutral *d*’ picture memory performance (C4: *r* = 0.59, *p* = 0.002). As such, more spindles were correlated with better next-day sleep-dependent neutral memory performance. We found no correlations with SO-spindle coupling density in the zolpidem condition. We did not find any correlations between spindle density or slow oscillation density and memory performance in the placebo condition.

## Discussion

We investigated the impact of zolpidem administration on emotional memory consolidation over a night of sleep. To this end, we used a double-blind, placebo-controlled, within-subject, cross-over design. We found that nighttime zolpidem administration reduced the time spent in REM sleep, increased the amount of time spent in NREM SWS, and increased fast sigma activity, whereas placebo showed greater N2 slow sigma and theta activity. In conjunction, we also found that in the zolpidem condition, subjects with worse negative picture performance at test 1 had subsequently greater relative SWA at night while those with better performance had subsequently greater relative sigma activity. Additionally, greater spindle density was correlated with the retention of neutral pictures. No relationships were found between sleep features and memory in the placebo condition.

The role of sleep in emotional memory consolidation has been the source of many investigations and little consensus. Early investigation into sleep-dependent emotional memory consolidation focused on the reciprocal role of REM in strengthening emotional memory details while minimizing reactivity (Payne & Kensinger, 2010; Wagner et al., 2001, 2005, 2006; Baran et al., 2012). Disruption of early SWS-rich sleep with the maintenance of later REM-rich sleep strengthened emotions, compared to neutral memories (Payne & Kensinger, 2010; Wagner et al., 2005, 2006). Supporting studies found that prefrontal theta activity during REM correlated with emotional memory (Nishida et al., 2009). However, emerging literature has also found that sleep benefits both negative and neutral memory retention and, more so, that REM correlates more with changes in arousal than actual episodic details of the memory. Converging on this finding and consistent with our prior nap study, we did not find a correlation between time spent in REM and subsequent emotional sleep-dependent memory consolidation for either sleep group (Kaestner et al., 2013). With zolpidem administration, our subjects in fact spent less total time in REM sleep yet continued to show enhanced negative memory. Moreover, zolpidem increased NREM slow-wave sleep and fast spindle activity in both stage 2 and stage 3. Our sleep feature analyses further revealed that in the zolpidem condition, poorer test 1 negative picture memory performance was correlated with subsequently greater relative SWA at night in stages 2 and 3, but greater test 1 negative picture memory performance was correlated with subsequently greater relative sigma activity. Additionally, the next day, test 2 neutral *d*’ picture memory performance was correlated with greater C4 spindle density. Analyses did not reveal relationships between sleep features and memory performance in the placebo condition.

Our findings converge with Cairney et al.’s (2015) null results comparing sleep and wake on negative and neutral pictures. However, the authors did report an inverse correlation between time spent in SWS and hippocampal activity during negative picture recognition. They concluded that this relationship was consistent with SWS’s contribution to system-level consolidation of negative emotional memories, with less reliance on the hippocampus for successful recognition after sleep. Similar to our paradigm, they tested memory after 12 h of wake and then again after a night of sleep. Thus, subjects’ emotional and neutral picture learning was subject to deterioration across wake prior to the nights’ sleep, while sleep protected and stabilized new memory traces (Feinberg et al., 2000; Rasch & Born, 2013). In both the placebo and zolpidem sleep conditions, the pictures learned in the morning underwent forgetting across the day prior to test 1 and nighttime sleep. This suggests that zolpidem may rescue deteriorating emotional memories by bolstering the negative content.

In general, it is suggested that emotional memories are longer lasting, more durable, and more resistant to forgetting than neutral memories (Payne & Kensinger, 2010; Wagner et al., 2006; Wagner et al., 2001 Wagner et al., 2005; Diekelmann et al., 2009; LaBar & Cabeza, 2006). On the one hand, this aligns with the literature that suggests sleep promotes the consolidation of stronger memories. Encoding of stressful and emotional events causes the release of norepinephrine and cortisol and engages the amygdala and tags the memory for further processing (Kobayashi et al., 2007; Payne & Kensinger, 2010). This is posited to prime subsequent sleep to preferentially consolidate the emotional memory trace via increased co-activation of the hippocampus and amygdala. This co-activation could increase the likelihood of hippocampal replay, enhancing the sleep-dependent memory consolidation for already strongly encoded memories (Girardeau et al., 2017). On the other hand, another literature body suggests that sleep should benefit more weakly encoded information, bolstering its resistance to future forgetting (see Diekelman et al., 2009 for review). In this way of thinking, sleep would benefit neutral more than negative memories due to their reduced saliency at encoding. Some studies have supported this alternative viewpoint, finding no benefit for emotional compared to neutral stimuli across sleep (see Lipinska et al., 2019 for review).

Our own study suggests a compromise between these two viewpoints. After a period of wake deterioration, the initially, more strongly encoded negative, not neutral, memory was rescued by zolpidem-induced sleep. However, in both conditions, the weakly learned, neutral picture memories were maintained. We also found a significant correlation in the zolpidem condition between the retention of neutral images and spindle density. This suggests that sigma activity may support the retention of neutral pictures.

Thus, we find enhancement and bolstering of negative information under zolpidem-induced sleep have important consequences for real-world experienced trauma. Psychological disorders associated with negative memories often have high reports of disturbed sleep and are frequently prescribed sleep aids (Colvonen et al., 2019; Ohayon & Shapiro, 2000; Peterson & Benca, 2006). After experiencing a trauma, increased sleep disturbances including insomnia and nightmares are risk factors for the development and maintenance of post-traumatic disorder (PTSD; Spoormaker & Montgomery, 2008; Ohayon & Shapiro, 2000). Interestingly, polysomnographic sleep architecture differences have not been consistently evident in the PTSD literature, with some studies pointing towards changes in SWS and others to REM shifts. One prior meta-analysis identified that overall, patients with PTSD spent more time in stage 1, less time in SWS, and had greater REM density (rapid eye movements/time spent in REM) than non-PTSD patients and controls (Kobayashi et al., 2007). Increased REM density has been linked to increased arousal and may be suggestive of poorer REM functioning (Barbato et al., 1994). More recently, high-density EEG was used to compare the sleep of combat-exposed soldiers diagnosed with and without PTSD (Wang, Laxminarayan, Cashmere, et al., 2020; Wang, Laxminarayan, Ramakrishnan, 2020; Wang, Ramakrishnan, Laxminarayan, 2020). Comparison of their sleep found that those with PTSD had reduced delta power over the central-parietal region, increased gamma power in NREM and REM over the anterior-frontal regions, higher frequency slow-spindles, and increased intra- and inter-hemispheric EEG synchrony in the alpha band (Laxminarayan et al., 2020; Wang, Laxminarayan, Cashmere, et al., 2020; Wang, Laxminarayan, Ramakrishnan, 2020; Wang, Ramakrishnan, Laxminarayan, 2020). With regard to pharmacological treatment, a case report study showed that administering zolpidem improved sleep disturbance symptoms while a more recent psychopharmacological treatment review did not show any symptom benefit (Dieperink & Drogemuller, 1999; Lipinska et al., 2016). The effects of zolpidem on the persistence of traumatic memories are concerning given that our results show that despite less time spent in REM, zolpidem bolstered a deteriorating negative memory.

Since prior emotional research has demonstrated that negative emotional memories can last years if sleep occurs shortly after learning, one could posit that the translation implication of administering zolpidem after a traumatic experience could create a memory resistant to decay (Wagner et al., 2005, 2006). How this decay-resistant memory impacts long-term clinical symptoms is unknown. On the one hand, heightened accuracy for traumatic or negative experiences could result in easier memory reactivation, leading to enhanced physiological and psychological distress. On the other hand, a sufficient level of detail may be required to support evidence-based therapeutic trauma interventions in which safe re-exposure and reframing of the content supports long-term mental health improvement. As Walker & van der Helm (2009) proposed, sleep should support the retention of the memory content, while separating the affective component; however, how the long-term strength of the content influences clinical symptoms is still unclear. As zolpidem appears to alter the typical trajectory for emotional memory retention, further research into the long-term repercussions of zolpidem on emotional sleep-dependent memory consolidation is needed, along with a more critical consideration of comorbid psychological disorders when prescribing this medication. Our study showed that zolpidem facilitated negative emotional memory consolidation among healthy adults without sleep disorders, which provides valuable information on the possible adverse effects of zolpidem on patients with mood disorders. Future studies are needed to see if zolpidem alters emotional memory consolidation similarly for patients with insomnia and mood disorders, which would have significant clinical implications.

### Limitations

A separate line of emotional memory consolidation research has demonstrated that REM sleep reduces the emotionality (physiological arousal) of a memory (van der Helm et al., 2011). In our study, we only evaluated the retention of emotional memory and thus cannot speak to changes in subjective arousal level when viewing the stimuli after sleep and drug administration. Given that we found less time spent in REM sleep with zolpidem ingestion, it remains an open question as to whether our subject’s subjective arousal would have changed. Unexpectedly, we found in the zolpidem condition that those with initial poorer negative test 1 memory had greater SWA during the night while those with better negative test 1 memory had greater sigma. Further research is needed to clarify this relationship. Additionally, in our study, we only evaluated memory performance change over a single period of sleep and thus cannot account for the morning learning rate in the sleep condition. Lastly, although we counterbalanced condition, picture set, and randomized valanced picture presentation, there is the potential that prior knowledge of the task could influence subsequent task motivation and encoding.

## Conclusion

Our study provides important evidence for understanding the mechanisms of sleep-dependent emotional memory consolidation. Zolpidem, a commonly prescribed sleep aid, has been shown to enhance sleep features associated with episodic memory consolidation and hinder forgetting of negative compared to neutral memory details. Furthermore, this bolstering of the negative memory occurred after a 12-h period of wake, suggesting that not only does zolpidem enhance emotional memory consolidation, it may rescue negative memories from typical forgetting and enhance their durability. Given that sleep difficulties are frequent symptoms of psychological disorders, such as post-traumatic stress disorder, our findings suggest caution in prescribing this specific sleep aid as the consequences of a potentially decay-resistant memory on long-term clinical symptoms are unknown.

## Supplementary Information

Below is the link to the electronic supplementary material.

Supplementary file1 (DOCX 57 KB)

## Additional Information

### Data Availability

Data are available at:  HYPERLINK “https://urldefense.com/v3/__https://osf.io/adhxb/__;!!LQC6Cpwp!8Zu7qVRL345rToMrgrmLnil7w8esIH0gAQmo-ihXbQEu66Ss-Uy8O-Kd5m6XO3AfmzE$” https://osf.io/adhxb/.
