# Supplementary material for: Zolpidem Maintains Memories for Negative Emotions Across a Night of Sleep
Source: Affect Sci. 2021 Nov 12;3(2):389–99. doi: 10.1007/s42761-021-00079-1 (PMC9249708; doi:10.1007/s42761-021-00079-1)
Supplement: Supplementary file 1 — Supplementary file1 (DOCX 57 KB) [file 42761_2021_79_MOESM1_ESM.docx]

**Supplemental Results.**

Similar to evaluating relative spectral power between conditions, we also evaluated differences in absolute spectral power. We first compared SWA and delta in the averaged F channels separately for sleep stage 2 and 3 (see Supplemental Table 1). We found no significant differences in SWA or delta between conditions. We then evaluated zolpidem’s effect on slow sigma, fast sigma, and theta in the averaged C channels separately for sleep stage 2 and 3. Both stage 2 and stage 3, compared to placebo, zolpidem showed significantly greater overall fast sigma in the averaged central channels (stage 2: t(27) = -2.176 *p* = .038; stage 3: t(26) = -2.164 *p* = .04). In addition, for Stage 2 in the placebo condition, subjects had greater central slow sigma (t(27) = 3.126 *p* = .004) and greater central theta activity (t(27) = 4.168, *p* < .001).
